# Sport and Dental Traumatology: Surgical Solutions and Prevention

**DOI:** 10.3390/dj9030033

**Published:** 2021-03-23

**Authors:** Lorenzo Mordini, Po Lee, Ricardo Lazaro, Roberto Biagi, Luca Giannetti

**Affiliations:** 1 Department of Periodontology, Tufts University School of Dental Medicine, 1 Kneeland Street, Boston, MA 02111, USA; Po.Lee@tufts.edu (P.L.); Ricardo.lazaro@tufts.edu (R.L.); 2 Department of Biomedical, Surgical and Dental Sciences, School of Dentistry, University of Milan, Via Della Commenda, 10-20122 Milan, Italy; Roberto.Biagi@unimi.it; 3 Department of Dentistry and Oral Maxillofacial Surgery, University of Modena and Reggio Emilia, Via Del Pozzo n°, 41-41124 Modena, Italy; luca.giannetti@unimore.it

**Keywords:** dental trauma, periodontology, dental implants, facial injury, tooth auto-transplantation

## Abstract

Trauma is a worldwide cause of millions of deaths and severe injuries every year, all over the world. Despite the limited extension of the oral region compared to the whole body, dental and oral injuries account for a fairly high percentage of all body traumas. Among head and neck traumas, dental and facial injuries are highly correlated to sport activities, and their management can be a real challenge for practitioners of any specialty. In case of trauma directed to periodontal structures, restorative and endodontic solutions may not be sufficient to achieve a definitive and long-lasting treatment. This article aims to illustrate surgical options and appliances to prevent dental injuries that may be available to the clinicians treating dental trauma involving oral soft and hard tissues.

## 1. Introduction

Every year, trauma is a worldwide cause of more than 5 million deaths for individuals up to the age of 45 years, causing half of all deaths in the age range of 10–24 years [1]. 

Despite the oral region representing 1% of the human body, injuries occurring in the oral region reach 5% of total bodily injuries among all ages, as shown by a one-year longitudinal prospective Swedish survey [2]. In Swedish newborn children up to 6 years of age, injuries to the oral cavity reach 17% of all bodily injuries. Due to their behavioral inclination, the oral region is at a higher risk in children and adolescents, compared to adults and elderly. In fact, maxillofacial trauma represented 33% of all types of trauma, as reported from hospital accident and emergency departments [3]. 

Dental and facial injuries are highly correlated to sport activities. Unfortunately, there is a high disparity of dental trauma definitions among scientific literature, which makes it difficult to outline an overall true prevalence [4]. As one could imagine, it has been reported that trauma as a result of sporting activities represents up to a third of all orofacial injuries (31%) [5,6]. In particular, 50.1% of those are traumatic dental injuries (TDI) [5,6]. Among contact sports athletes, the prevalence of TDI varies between 7.1% and 71.5% [7]. Despite the significant variation, studies have indicated that the prevalence of trauma is less than 40%, depending on the type of sport practiced [8]. In other words, the incidence varies between the different types of sport. The most common TDI occur in the maxillary incisors, accounting for 80% of all cases [9,10]. Among patients reporting history of injuries to the oral region, 92% presented with dental trauma, 28% soft tissue injuries and 6% with bone fractures [2]. It was not uncommon to see combinations of the above. 

When injuries are restricted to the soft tissues, such as lacerations, abrasions, and contusions [11], they create wounds that usually heal without major complications. However, trauma to the dentofacial structures might result in serious injuries that often require tooth extraction, bone regeneration, and prosthetic replacements [4]. The consequences of these injuries can potentially cause severe pain, emotional and psychological impacts, as well as economic implications [12]. In fact, a study reported that the mean cost of maxillofacial and dental injuries was more than double that of all other bodily injuries occurred in contact sports [13]. When the dental trauma extends to the supporting periodontal apparatus, more extensive treatment may be required. The intervention of a specialist may be needed to restore not only dental structure but bone and soft tissue that was damaged during the injury. 

This article aims to illustrate surgical options and the appliances to prevent dental injuries caused by sport activities; a review of techniques that may be available to the clinicians treating dental trauma involving soft tissue injuries and alveolar bone will be presented.

## 2. Dental Trauma and Injuries

### 2.1. Traumatic Dental Injuries (TDI)

Traumatic forces are considered one of the four most frequent oral diseases [14]. They not only affect tooth structure, but they can disrupt the supporting periodontal apparatus, including bone and peripheral soft tissues. TDI related to teeth include crown and/or root fracture involving the pulp or not. Depending on the magnitude of the injury, teeth can also experience different degrees of periodontal support alteration, such as concussion, sub-luxation, luxation, and avulsion [14]. Their specific definitions are listed in Table 1.

### 2.2. Prevalence

#### 2.2.1. Prevalence Contact Sports

Contact sports are considered activities which aim is to utilize physical contact to lead the team or the individual to win a competition. The physical contact during these competitions is intense and, competitors a have a high risk for dentofacial injuries. 

A systematic review and meta-analysis of 17 articles [17] showed a total prevalence of dentofacial injuries of almost 30%. When single sports were evaluated, rugby presented a prevalence of almost 40%, basketball 27.26%, handball 24.59%, and field hockey 19.07%. Among all injuries, the most common was dental trauma (19.61%). The main limitation of this study was the heterogeneity within the selected studies. The major risk of bias of the studies reported in the systematic review was the small sample sizes, not ideal for prevalence studies (≤400). For this reason, it was not possible to generalize the prevalence in the general population. The same study reported that men under 30 who play sports for at least 4 h a week, have the highest risk of injuries related to sport [17].

It is interesting to know that there is no mandatory use of mouthguards for contact sports in USA. 

The American Dental Association recommends the use of mouthguards for many sports, including basketball, martial arts, boxing, rugby, football, soccer, hockey, wrestling, lacrosse, and many others [18,19], but only the National Federation of State High School Associations (NFHS) [20] mandates the use of mouthguards for football, hockey, lacrosse, and wrestling (only if wearing braces).

These high data on prevalence are concerning, and they show the magnitude of dental injuries caused by sport activities. The general population can be affected by esthetic and functional issues, while professional athletes can see a reduction in their performances and participation to games and matches. Data showed that ~60% of injuries caused forced inactivity or occupational disability, damaging athletes’ activities and profession [21].

#### 2.2.2. Prevalence Combat Sports

**Figure 1:**
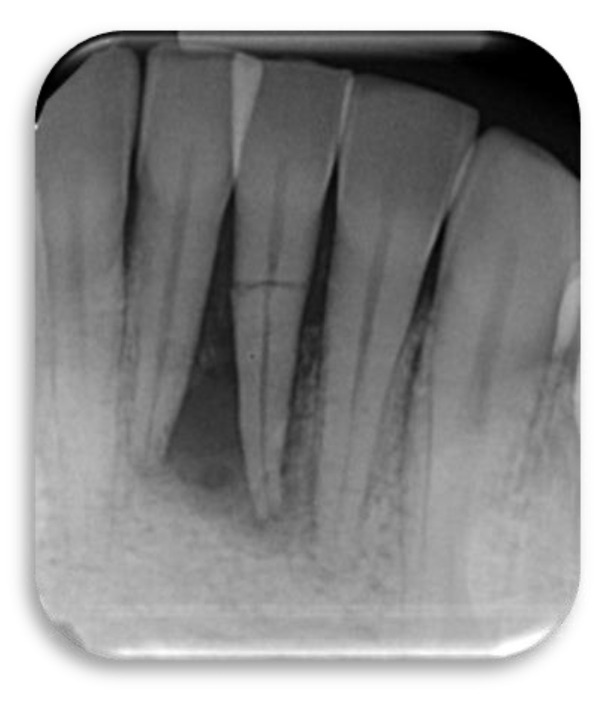
This radiograph shows tooth#24 horizontal fracture and radiolucency in the anterior mandible, as a result of a blow received from the patient playing boxing.

Many are the sports that can be listed in the “combat” category: boxing, judo, karate, kendo, kung fu, taekwondo, muay thai, sumo, capoeira, fencing, jiu-jitsu, wrestling, and wushu are the most famous around the world (Figure 1).

Results from a recent systematic review and meta-analysis suggested a dental injury pooled prevalence of ~25% and dentofacial injury pooled prevalence of 30.3% [7]. Individual sport analysis showed jiu-jitsu to have the highest combined prevalence of dentofacial injuries (52.9%), while judo was the lowest (25%). Among sports that are popular in the Americas, boxing and wrestling had the highest prevalence of dental injuries, reaching up to 80.0% in some studies [7]. It has to be noted that the heterogeneity on the estimated prevalence is high. Some studies obtained data from questionnaires on lifetime past history while only few studies considered a specific time (from 1 to 15 years) [7]. This systematic review and meta-analysis included papers with different population background and skill level, such as competitors, non-, semi- and professionals, and elite athletes. As it can be expected, professionals and competitive athletes showed higher prevalence of dentofacial injuries compared to non-professional ones. In fact, these top competitors reach higher intensities during competitions and training. A role may be also played by higher responsibilities and pressure deriving from sponsors and awards. Only one study reported the highest prevalence rate of 41.4% of the African continent. The American continent ranked second for dentofacial and dental only injuries [7]. Once again, heterogeneity at various levels represented a limitation. Age groups were highly heterogeneous, and some athletes may have performed in different combat sport; both these aspects may have influenced the real total prevalence. Despite lack of description regarding the type of dentofacial injuries reported, tooth fracture was the most common (6% to 50%). With regard to dentofacial trauma, malar bone contusion (0.71–11%) and labial laceration (11–15%) were the most prevalent [7].

### 2.3. Epidemiology

In most of the studies, boys experience dental traumas more often compared to girls, to ratios that reach up to 2.5:1. Boys’ permanent teeth are affected almost twice than girls’, most likely because they participate more actively and have more propensity for contact and combat games and sports [14,22,23]. In the latest years this trend has been levelled by girls’ increased participation and competitive behavior in sports like hockey and soccer that were once regarded as “boys’ games”, especially modern Western society [14,24]. Data from literature [14] demonstrated that most dental trauma occur during childhood and adolescence. It is estimated that up to 90% of all dental injuries sustained in a lifetime occur before the age of 19 years. Data showed how traumatic tooth injuries decrease after the twenties. The higher risk of dental injuries among children that belong to higher socio-economic backgrounds, may be due related to easier access to bicycles, skiing, skateboards, horse-riding, and swimming pools than those from low socio-economic groups [14].

### 2.4. Teeth Involved

Not all individuals experiencing sports-related dental trauma are affected in the same way. Some dental malocclusions (class II) are significant predisposing factors, such as increased overjet with protrusion of upper incisors and insufficient lip closure [14]. The World TDI prevalence in primary dentition, of patients up to 5 years of age, is 23% (17.3–29%); the prevalence on permanent dentition on patients younger than 30 years of age is 15.5% (13.2–17.9%), with significant decreases afterwards [14].

The preponderance of dental injuries involves the anterior sextants, in particular the maxillary central incisors, while the maxillary lateral and mandibular central incisors are involved to a lesser degree. Dental injuries occurring during sport events, can result in a single or multiple tooth injuries, especially among teenagers [14].

### 2.5. Etiology

Generally speaking, dental traumas are caused by a collision that can generate a high energy force leading to an injury. This can derive from an object used for sports (ball, stick, car) or from another athlete’s body part or animal (horse hoofs). Depending on the entity and object of the force, the dental injury can be limited or extended. Physical leisure activities can be associated to sports on their likelihood of dental injuries [14]. For instance, bicycling and skateboarding are the second most frequent injury cause among adolescents, being responsible for almost 20% of all traumatic events [14]. Sport is responsible for injuries to permanent teeth, accounting for 13% of all injuries (Table 2).

## 3. Treatment Options

Depending on the extent, severity and location of the injury, different treatment options may be needed. In the mild traumatic events, the amount of force received leads to a simple enamel craze lines or a small size enamel-dentin fracture. These cases are treated with minimal invasive restorative procedures and do not require any surgical intervention [14]. Nevertheless, dental trauma does not only affect tooth structure but can alter, interrupt or permanently damage the periodontal ligament and attachment apparatus, leading to dental, soft and hard tissues.

**Figure 2:**
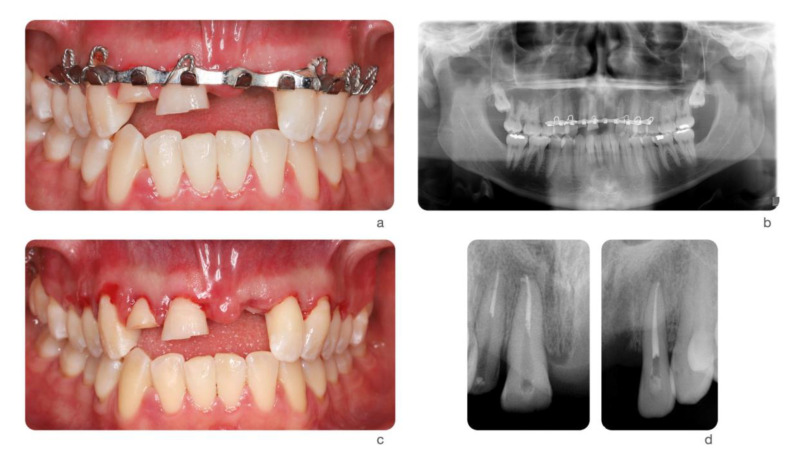
Ski accident that involved a 26-year-old male. He was treated in the emergency room with a metal retainer anchored with orthodontic wire around teeth involved in the trauma (**a**). The diagnosis was non-complicated maxillary fracture and teeth #7, 8, and 10 concussion and #9 avulsion. Panoramic image of the metal retainer (**b**). After 1 month of healing, metal retainer was removed (**c**) and teeth #7, 8, and 10 diagnosed as necrotic. Root canal treatments were performed (**d**).

Different sports require different healing time. In non-combat sports, the return to play at a professional level can be as fast as one to two weeks for an isolated fracture, but multiple fractures may require a longer period (Figure 2). Combat athlete may be stopped for longer time in order not to compromise the healing. To date, there is no clear evidence-based guidance [25].

The following injuries are usually treated routinely in a dental office by either the restorative dentist or surgeon.

### 3.1. Tooth Avulsion

Avulsion is the most impacting dental trauma event; its emergency treatment is crucial for the fate of the tooth involved. Avulsion can be defined as the complete displacement of the tooth out of its socket. All avulsed permanent and mature teeth eventually develop pulp necrosis [26]. While revascularization could be achieved in teeth that did not complete their root maturation, but success rates are not higher than 50% [14,26,27]. Studies have indicated that early replantation is critical for the best chance of success [26,27,28] (Table 3).

There are three possible clinical scenarios to treat avulsed permanent teeth: (1) The tooth has been replanted at the site of injury or before the patient’s arrival at the dental clinic. (2) The tooth has been kept in a physiologic storage medium or stored in non-physiologic conditions, with the extra-oral dry time being less than 60 min. (3) The tooth has been in extra-oral dry time longer than 60 min [29]. The 60 min extra oral time had been identified as the threshold after which all periodontal ligament cells undergo necrosis and replacement resorption [30,31]. The reason could be identified in a pulpal bacterial contamination leading to an inflammatory resorption in association with a periodontal ligament damage. The critical time varies between studies but many authors consider an extraoral dry time of 15 min or less to reduce resorption [32].

1. If the tooth has already been replanted, the injured area should be cleaned with water, saline or chlorhexidine. After, the correct position of the replanted tooth should be verified clinically and radiographically. The tooth should be left in place, except if the tooth was positioned in the wrong position. In that case, it should be corrected with slight finger pressure. Local anesthesia should be administered, if necessary, and preferably with no vasoconstrictor to presence the vascularity. If the teeth were replanted in the wrong socket or rotated, the tooth should be repositioned up to 48 h after the traumatic incident. The tooth should be stabilized for 2 weeks using a passive flexible splint such as wire of a diameter up to 0.4 mm or nylon fishing line. The composite and bonding agents should leave a hygienic space, with some distance from from the gingival tissues and interproximal areas. In cases of associated alveolar fracture, a more rigid splint is indicated and should be left in place for about 4 weeks. Finally, gingival lacerations, if present, should be sutured and systemic antibiotics should be prescribed [29].
2. In the other two scenarios, the root surface should be rinsed with saline or osmolality-balanced media to remove gross debris by gently agitating it in the storage medium. the socket should be irrigated with sterile saline. If there is a fracture of the socket wall, the fractured fragment should be repositioned into its original position. The removal of the coagulum with a saline stream may allow better repositioning of the tooth. the tooth should be slowly replanted with slight digital pressure [29].

In all situations, root canal therapy should be initiated within 2 weeks following replantation. Clinical and radiographic examination should be carried out at 2 weeks, 1 month, 3 months, 6 months, and yearly, at least for 5 years. First-aid actions have to be promoted among the general population as the prognosis of the tooth is extremely related to the actions taken at the place of the accident [15].

When the tooth is immature and presents an open apex, the goal is to achieve pulp revascularization, which can lead to further root development. The risk of external root resorption should be weighed against the chances of revascularization as the resorption is very rapid in children. If spontaneous revascularization does not occur, apexification, pulp revitalization/ revascularization, or root canal therapy should be initiated as soon as pulp necrosis and infection is identified [29].

### 3.2. Auto-Transplantation

Tooth auto transplantation (TAT) was first proposed in 1970s as a predictable treatment to restore missing tooth for its long-term success [33]. The potential benefits of this approach were well-documented in literature. TAT can be performed on growing individuals with open-apex teeth; it can retain the regenerative potentials for alveolar tissues at recipient site; moreover, the transplanted teeth can further erupt to achieve the harmonic periodontal and occlusal stability [34]. Although the protocol of TAT was initially established for tooth with incomplete root formation (Ideally, 1/2 to 3/4 of expected complete root length), several studies have shown the successful long-term outcomes for teeth with both complete and incomplete root formation [34,35,36,37]. Specifically, according to meta-analysis by Chung [37], the 1-year and 5-year survival rate of close-apex TAT are 98% and 90.5%, respectively. On the other hand, according to meta-analysis by Atala-Acevedo [36], the survival rate of open-apex TAT is 98.21% with mean follow-up of 6 years. However, the results should be interpreted with caution since this surgical approach is highly technique-sensitive and requires strict case selection. Two main possible post-operative complications are reported as root resorption and ankylosis. They are commonly resulted from the inflammatory response to damage of periodontal ligament on donor teeth and the following tissue repair mechanism. Although the prevalence of root resorption and ankylosis varies among studies, it was concluded as 4% and 4.8% respectively in meta-analysis by Machado [34].

Regarding surgical steps, there are several factors which may influence the clinical outcomes and therefore need to be taken into consideration in treatment planning. According to meta-analysis by Chung et al. [37], the rate of root resorption is two times higher on transplanted with endodontic treatment after 14 days postoperatively than within 14 days postoperatively. Secondly, the estimated failure rate is higher in transplanted teeth with splinting within 14 days postoperatively than after 14 days [37]. The use of systemic antibiotics shows clinical benefit of reducing failure rate and rate of root resorption. Interestingly, it is shown that molar donor teeth have lower rates of failure and complications. The reasons could be attributed to the larger surface area of periodontal ligament and higher loading of masticatory functions. However, due to insufficient well-controlled clinical trials, data remain inconclusive [37].

Due to the nature of traumatic dental injuries, the prevalence is higher is anterior teeth. Several clinical studies have shown the high successful rate of TAT from premolars to maxillary anterior site. Although it is relatively rare to assess patient’s perspective for the esthetic outcomes, several studies presented high patient-reported satisfaction after surgery [35].

**Figure 3:**
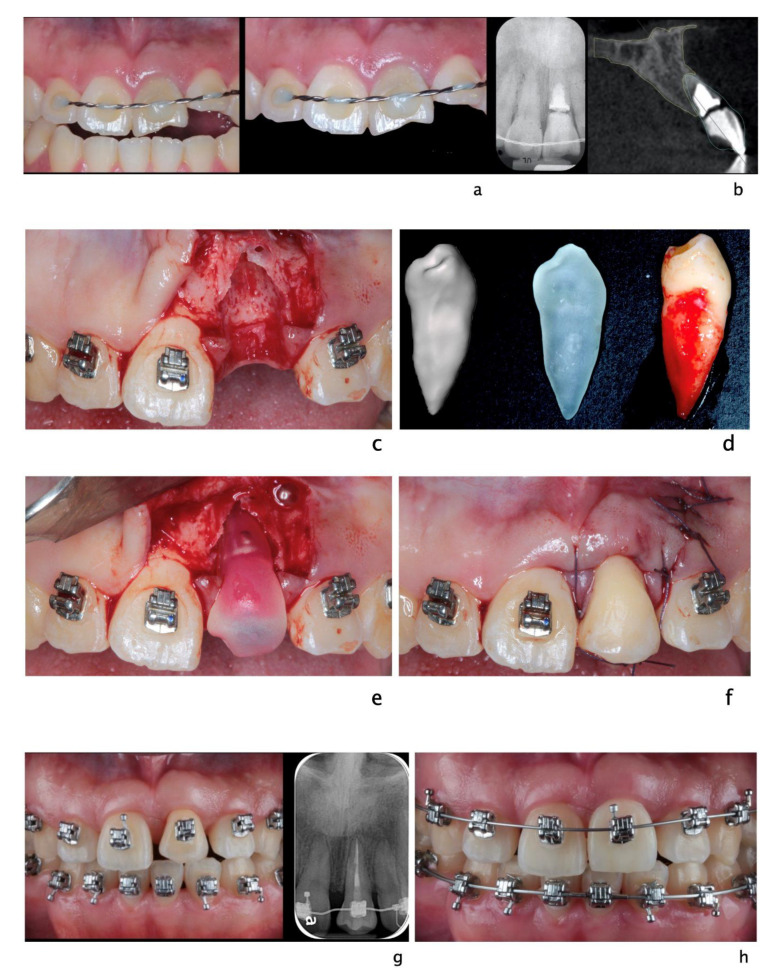
A 20-year-old Asian male had #8 diagnosed with a root fractured due to sport related trauma (**a**). The tooth was endodontically treated, followed by healing with interposition of connective tissue (**b**). After healing was completed, a second sport injury involved the same tooth. The tooth mobility increased, and a periodontal lesion was diagnosed by elevated probing depth. The tooth was stabilized with orthodontic wire and patient was referred to periodontist for evaluation. Combined with malocclusion and anterior open-bite, the treatment plan was made as full-month orthodontics and auto-transplantation of #28. Tooth #8 and 28 were extracted (**c**,**d**) and a premolar replica was printed (**d**). After socket adjustment with the replica (**e**), tooth #28 was stabilized in place with sutures (**f**). After periodontal stabilization and verification of periodontal healing (**g**), the final restoration was delivered (**h**).

From a clinical point of view, the success of TAT is influenced by several factors which rely on surgeon’s preoperative assessment, surgical skills and experience [38]. Technically, the more attempts of positioning the tooth correspond with more potential damages to the periodontal attachment apparatus. Nowadays, with the advances of dental cone-beam computed tomography (CBCT) systems and 3D printing technology, the surgeon is able to comprehensively evaluate the donor tooth and recipient extraction socket to plan their combination. The use of printed replica significant decreased the extraoral time of donor tooth during preparation. The prototyping-guided TAT was systematically reviewed [38]. The success and survival rates are 80.0–91.1% and 95.5–100% respectively [38], and generally the extraoral time during manipulation can be reduced to less than several minutes (Figure 3).

Auto-transplantations could be utilized where no adhesive prosthetic solutions could be delivered, patient’s refusal of mini-implant, and availability or teeth to be transplanted. Unfortunately, not all patients present available teeth to be implanted, and the site of transplantation may be severely damaged by the injury, impeding the acceptance of the replacing tooth [14].

### 3.3. Soft and Hard Tissue Reconstruction

To the best of the author’s knowledge, there are no articles proposing specific protocols regarding periodontal tissue rehabilitation after sport trauma. The soft and hard tissue deficiencies following trauma should be re-evaluated after initial healing and stabilization. The deficiencies then can be treated as clinical scenario requiring soft and hard tissue regeneration [39]. One of the consequences after dental traumatic injuries is bone resorption, resulting from either tooth luxation, avulsion or fracture of alveolar process [14]. Severe ridge deficiency may require multiple surgical inventions to regain esthetics and functions.

A recent systematic review and meta-analysis on lateral ridge augmentation [40] reported an estimated pooled mean bone gain at the time of regeneration of 3.71 ± 0.24 mm. Taking into account the physiological bone shrinkage, the estimated mean decrease after 6 months was 1.13 ± 0.25 mm. Regardless of the technique used for bone grafting, different degrees of graft resorption should always be expected. To compensate for this occurrence, overcorrection of the horizontal defects should be taken into consideration. Mordini et al. [41] showed 5% ± 3.78% resorption rates from 4 to 6 months after guided bone regeneration in posterior mandible affected by horizontal bone loss.

Elnayef et al. [42] analyzed the efficacy of vertical bone augmentation. The weighted mean was 4.49 ± 0.33 mm with no specific technique showing superiority in terms of implant survival and success rates. Guided bone regeneration showed fewest complications.

Periodontal soft tissue deficiency is also often seen after traumatic dental injuries [43]. With extensive damages to the gingival tissues, underlying periosteum and alveolar bone, unfavorable responses to soft tissues may occur. Especially under concomitant bacterial invasion or severe dysbiosis during wound healing phases, the “pink esthetics” may be significantly compromised. To enhance the periodontal soft phenotypes, to increase the width of keratinized tissues and to gain the root coverage for recession, several surgical approaches with different available materials have been purposed. A recent meta-analysis by Barootchi [44] concluded that autogenous tissue grafts seem superior compared to non-autogenous grafts for all procedures. However, non-autogenous grafts still offer as clinically effective options.

### 3.4. Dental Implants

**Figure 4:**
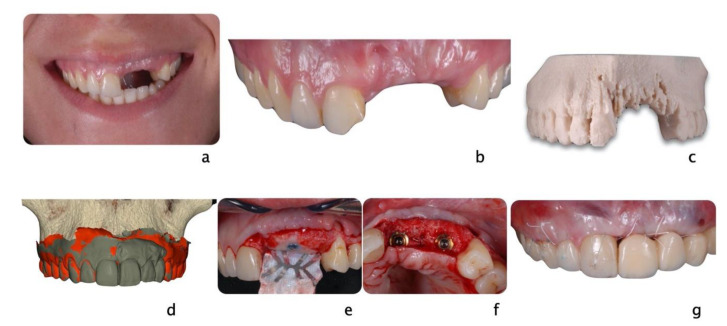
A 24-year-old female fell from her bike during a race. She hit the tarmac and resulted in losing teeth#9, 10 and 11 (**a**,**b**) as well as a portion of the alveolar bone (**c**) as seen on the 3D print of the maxilla. An incisal chip on tooth #8 completed the damage of the fall. After an analysis of residual hard and soft tissue volumes, a digital wax-up was created to plan the future implant placement and restorations (**d**). Guided tissue regeneration was performed, and implants were placed in a Type 4 timeline (**e**,**f**). A provisional fixed partial denture and connective tissue graft were inserted to improve esthetics and tissue conditioning (**g**).

TDI may lead to as series of events that include tooth avulsion or need for extraction. Many options are available for tooth replacement and one of these are dental implants. This device, used following tooth extraction, has been proven as a successful approach to restore function and esthetics after traumatic dental injuries. To optimize the outcomes of implant placement, there are several clinical parameters which should be taken into considerations in treatment planning (Figure 4). In clinical scenarios, the timing of implant placement associate with tooth extraction is generally categorized as [45]:

- Type 1, immediate placement, no later than 24 h after tooth extraction.
- Type 2, early placement, typically 4 to 8 weeks after tooth extraction with only healed soft tissue at extraction site.
- Type 3, early placement, typically 12 to 16 weeks after tooth extraction with healed soft tissue and significant healing of alveolar bone at extraction site.
- Type 4, late placement, after 6 months with complete healing at extraction site.

Following traumatic dental injuries like tooth avulsion, the considerations of implant placement can be made as for non-trauma related tooth extraction. The time period of the tooth outside the socket and the presence of damage on alveolar supporting tissues significantly influence the decision regarding timing of implant placement and the need for adjunctive procedure, i.e., tissue augmentation. In the very rare case scenario of immediate tooth avulsion with intact alveolar soft and hard tissues, when tooth replantation is not indicated, type 1 implant placement can be considered. The predictability of successful immediate implant placement relies on the initial thickness of buccal plate, soft tissue phenotype and primary stability provided by bone apical and palatal to the socket. Type 2 or 3 implant placement, referring to early placement with healed soft tissue and partial bone fill, is more relevant to dental trauma. Additional tissue augmentation is usually indicated to offer stable three-dimensional environment for ideal implant placement after traumatic dental injuries. Type 4 implant placement, as a delayed treatment, is indicated when the treatment plan involves the adjacent teeth for more complex clinical scenarios. Although there is limited specific clinical trial comparing different protocols of implant placement following traumatic dental injuries, evidence has shown that, if guidelines are strictly followed, different protocols offer similar successful outcomes [46,47,48].

### 3.5. Other Treatment Options

Due to the high prevalence of traumatic dental injuries in young population with ongoing craniofacial growth and development, clinician often face the decision whether or not to place implant to restore the missing teeth. The implant infra-position is the most pronounced risk after implant placement in this situation, especially in anterior maxilla. Moreover, the implant survival rate is lower in growing population compared to elder group [49]. Unfortunately, the degree of continuous growth varies among individuals and even people who had implant placement at middle ages experienced noticeable implant infra-position [50,51]. That is to say, judging the craniofacial grow pattern only by age is not fully accurate although it was reported that the rate of implant infra-position significantly decreases in population older than 30 years [52]. Although the craniofacial growth was reported as continuous development even after puberty, the amount of annual growth significantly decreased over time, especially after 20 years old [53].

**Figure 5:**
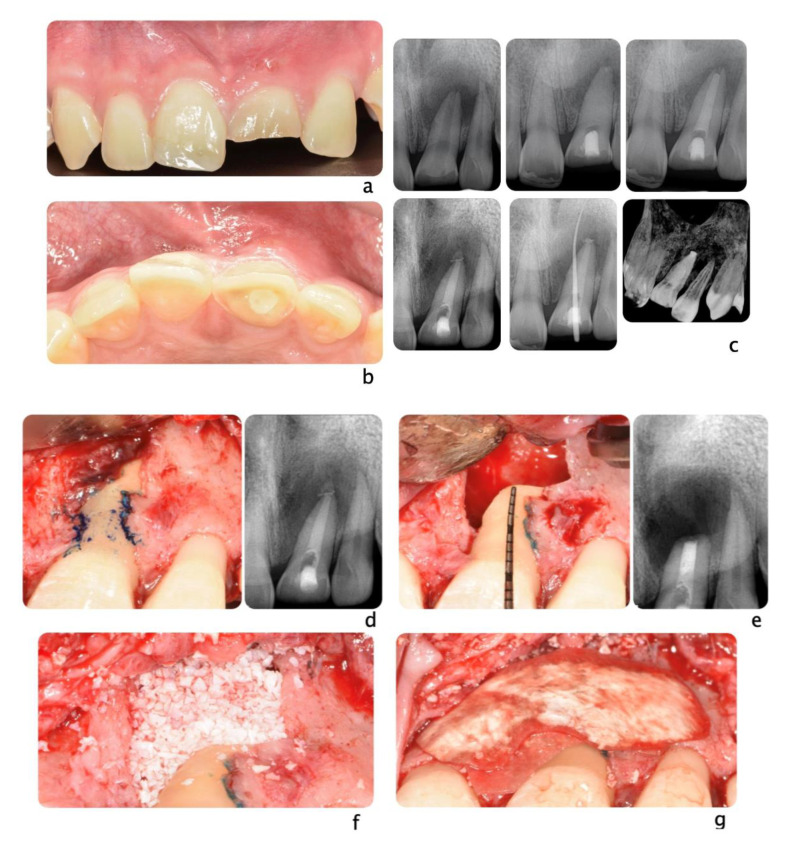

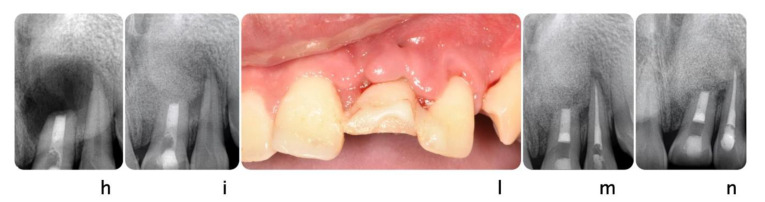
Clinical scenario of a 17 years-old adolescent hit by a baseball ball in the anterior maxillary region. The boy presented to the Periodontal Department at Tufts University, Boston USA with crown fracture of left central incisor (#9) (**a**,**b**). Peri-apical radiograph show apical radiolucency, sign of necrosis. After the diagnosis, CaOH_2_ was applied. The root canal definitive treatment was completed but after 2 months the patient still presented with a fistula, that was tracked via a gutta-percha point. A CBCT scan was performed in order to diagnose the extent of the peri-apical lesion (**c** in sequence). The extent of the lesion did not suggest an endodontic therapy revision. Exploratory surgery was performed in order to rule out tooth fracture (**d**). The apex was resected in order to access the palatal aspect of the tooth. A PA radiograph was taken in order to verify correct apex resection and endodontic retrograde seal (**e**). Due to active patient skeletal growth, a decision was made to enucleate the endodontic cyst and treat the cavity with bone grafting material, in order to preserve the site for future implant placement (**f**,**g**). PA radiograph comparison before and after grafting placement (**h**,**i**). The patient was followed up for 2 months, and a fistula was identified apical to #9 (**l**). Tooth #10 was diagnosed as necrotic. A root canal was performed (**m**) and the apical radiolucency and fistula were resolved at 1 month follow up (**n**).

Different approaches have been proposed to measure the cessation of craniofacial growth, such as hand-wrist radiograph and cephalometric analysis [54]. However, it was reported that hand-wrist radiographic measurement is not ideal at determining the cessation of craniofacial development due to low accuracy [55,56]. Therefore, in order to identify appropriate timing for implant placement in adolescents one should not rely on only one measurement or exam. Yearly lateral cephalometric radiographs should be taken to evaluate the continuous tooth eruption and skeletal growth [54]. Some clinicians proposed to place the implants in a more coronal or shallow position, to compensate the physiological adjacent natural tooth migration. However, there is lack of consensus due the diversity and individual growth pattern that cannot provide long-term predictability. The interdisciplinary collaboration in planning phase is of paramount importance. Once the decision is made to postpone the implant placement, temporalization is important to maintain the functional and esthetic demands. In case of injuries that impaired the available bone for future implant placement, soft and hard tissue augmentation is recommended in order to prepare the site for future implant placement (Figure 5).

Another technique that can be used in adolescents that did not yet complete skeletal growth, is the use of mini-implants. This technique exploits the advantages of conventional diameter implants and those of reduced diameter implants both during the positioning phases of the fixtures and during the prosthetic phases. A fixed rehabilitation provides an obvious psychosocial benefit especially during the complex adolescent age. At the same time, mini-implants can be replaced by a standard fixture upon completion of growth and increased esthetic demand from patients. In other words, the use of mini-implants in growing patients can be both used as a temporary and definitive treatment option. Therefore, the possibility of carrying out post-traumatic rehabilitation using mini-implants and cemented prosthetic crowns becomes an interesting alternative for the growing patient [57,58,59]. Yet, some concerns regarding the long-term survival rate of these implants still remain. In 2018, the ITI Consensus Report [60] evaluated the influence of implant factors on clinical and radiographic outcomes. One of the analyses regarded the influence of implant diameter, comparing three categories of narrow implants; category 1 or “Mini-implants” <2.5 mm; category 2: >2.5 mm and <3 mm; category 3: >3 mm and <3.5 mm). The mean survival rates resulted in 94.7% ± 5%, 97.3% ± 5% and 97.7% ± 2.3% for category 1, 2, and 3, respectively. The authors concluded that implants with diameters of less than 2.5 mm showed inferior survival rates compared to standard diameter implants. Similar results were obtained in a systematic review by Bidra et al. [61]. The authors evaluated the short-term (1 to 5 years), medium-term (5 to 10 years), and long-term (beyond 10 years) survival rates of mini-implants employed for final prosthetic treatment. The 1st year survival rate was <95% and the cumulative survival of 9 years was ~92%. Most implants were immediately loaded, and the majority of implant failures happened within one year of placement. The reported a one-year survival rate was considered acceptable but many implants analyzed had a minimum follow-up shorter than a year. The authors concluded that there were insufficient data regarding failures after the first year. It is not safe to draw conclusions regarding the 5–10-year survival rate of mini-implants. The authors could not find any evidence for the long-term survival of these implants.

## 4. Prevention

### Appliances to Prevent Dental Injuries

During sports events, the risk of falling, being hit by an opponent or a ball is high. The sole way of minimizing the number of TDI are to implement preventive approaches. The two main appliances to prevent TDI are faceguards and mouthguards. Prior to the mandatory utilization of face and mouthguards in American football, facial, and oral trauma accounted for 50% of all reported injuries [14]. With the introduction of these protective gear, the incidence of oral and facial trauma significantly decreased down to a 3.9% [62]. The use of mouthguards decreases 5.55 times the chance of players suffering dental injuries [63]. 

Faceguards are prefabricated or custom-made cages made of metal, composite or, more recently, clear polycarbonate plastic which are usually attached to helmets or head straps. Faceguards and helmets have been effective in practically eliminating all oculofacial injuries in contact sports. Full face shields have demonstrated to significantly reduce the risk of dento-facial injuries without increasing the risk of neck injuries or concussions [14].

Mouthguards are considered the main appliance for the prevention and reduction of severity of sports-related oral injuries. A mouthguard is defined as a resilient device placed inside the mouth in order to protect the player [64]. They were introduced in the 19th century by a dentist named Woolf Krause and their main intention was to protect boxers from soft tissue lacerations [65].

Mouthguards can prevent bruising and lacerations of the perioral and intraoral tissues, protect teeth from avulsion, luxation, crown or root fracture, avoid jaw fractures and dislocations, provide support for the edentulous space, and minimize the severity of condylar dislocation and temporomandibular joint trauma [66]. A systematic review reported that athletes wearing mouthguards are 82% to 93% less likely to suffer TDIs [8]. In fact, the prevalence of dental trauma ranged from 7.5% to 7.75% among mouthguard wearers compared to 48.31% to 59.98% for non-wearers [8]. Although it was noted that avulsions and crown fractures were the most frequent injuries [8], overall the number of reported tooth fractures has been significantly reduced with the use of mouthguards [14]. A meta-analysis carried out in 2020, indicated that athletes involved in many different sports that were not wearing mouthguards showed twice higher risk of suffering orofacial injuries compared to those wearing one [67]. In a prospective longitudinal study analyzing 70,936 athlete exposures, mouthguard users had significantly lower rates of TDI—fractures, luxation and avulsions—(0.6% vs. 2.0%) and lower dental referrals (1.6% vs. 5.8%) compared to non-users [68]. Only one study did not find statistically significant differences in terms of head, neck, and oral injuries between users and non-users of mouthguards [69].

The main four purposes of mouthguards that have been described in the literature are: (1) Protecting teeth by absorbing or dissipating the energy of a shock. (2) Preventing lacerations on lips, tongue and gingival tissues. (3) Protecting antagonist teeth from traumatic occlusal contact. (4) Providing resilient support to the mandible by absorbing impacts that could fracture the angle or condyle of the mandible.

A fifth proposed function is the protection against neck and cerebral injuries. However, studies still haven’t been able to demonstrate it [68].

The mechanism in which mouthguards achieve their functions is still not clear. Hypothesis have been formulated that the higher the absorption energy, the higher the protection. Nevertheless, this absorbed energy may be transmitted to the underlying dental and periodontal tissues [70].

There are 3 main types of available mouthguards: 1. Stock (commercially prefabricated mouthguard sold over the counter with a claimed universal fit and designed to be used without modification) 2. “Boil-and-bite” (commercially available mouthguard that is made of a thermoplastic material that is softened in hot water and the athlete adapts it to their own dental arch using finger, tongue, and biting pressure) 3. Custom-made (made by a dentist and dental technician using the patient’s cast) [71].

Both stock and boil-and-bite mouthguards have been reported to lack of proper retention and require the user to apply constant occlusal pressure to held them in place making them uncomfortable. Custom-made mouthguards are tailor-designed to provide better fit and comfort as they allow for easier and better breathing and speaking [71,72,73]. Although further comparative studies with larger sample size and longer follow-up time are need, some studies suggest that custom-made mouthguards offer better protective characteristics [70]. Increased comfort of wear can be experienced if the mouthguard is extended labially to within 2 mm of the vestibular fold as extended as far as the patient can tolerate it and adjusted to allow clearance of frenum [74].

Mouthguards should extend at least up to the distal aspect of the maxillary first molar and should have a labial and occlusal thickness of 3 mm and a palatal thickness of 2 mm. to reduce the effects of impact force on teeth. Occlusion of mouthguards should be bilateral and balanced [75]. The reduction of the palatal extension of mouthguards from 6 to 2 mm. improves the degree of satisfaction of athletes [76].

Custom mouthguard should follow some specific criteria such as being made of an easily cleaned and disinfected, resilient material, and provide correct retention allowing an adequate occlusal relationship and maximum protection [14]. Furthermore, they should be able to absorb and deflect the energy of an impact by covering the maxillary teeth, exclude any occlusal interference, allow mouth breathing, and protect the soft tissues.

Mouthguards offer a considerable protection against sports-related dental injuries and custom-made mouthguards provide advantages over the other types. Lately, athletes are becoming more aware of the importance of using mouthguards as preventive measures to avoid orofacial injuries. In a recent study, 95.7% of a sample of 310 hockey players reported to have tried a mouthguard while 86.8% and 91.3% used them regularly during training sessions and competition, respectively [77]. However, other studies have observed that there is still a high number of athletes and sport players that do not use them because of the perception of being expensive and the need of at least one visit to the dentist [71]. It is important that members of the technical staff encourage the regular use of mouth guards in athletes who practice risk contact sports [71,78,79].

Dentists should also promote the use of mouthguards among professional and amateur athletes. Nevertheless, the lack of evidence-based guidelines has reflected the different perspectives and beliefs of orthodontists about recommending the use of mouthguard [80]. During the past years, there has been an exponential increase in the number of athletes worldwide and dental trauma has become a significative dental health issue. In 2016, April was established as the National Facial Protection Month. During this month five different health organizations (American Academy of Pediatric Dentistry, the Academy for Sports Dentistry, American Association of Orthodontists and the American Association of Oral and Maxillofacial Surgeons) promote orofacial protection and the use of mouthguards [81].

It has been shown that prevention is the most effective way of decreasing TDIs and, for this reason, sports committees and public authorities should regulate and establish mouthguards as mandatory safety equipment that protect the physical integrity of athletes [8].

## 5. Discussion

As reported in the previous paragraphs, sport injuries can lead to extensive and debilitating fractures that can involve both facial and dental structures. Many challenges arise for the clinicians in charge of handling these patients. Many parameters and therapeutic options need to be taken into consideration to successfully treat and resolve these compromised cases [82]. Patients’ age, their medical history and compliance need to be carefully reviewed in order to outline the most ideal treatment planning. Nevertheless, even taking into account all these variables, surgical solutions may be comparable, making the clinical final decision not straight forward [82].

Taking into consideration both patients to the site of injury, some of the technical considerations that need to be evaluated are the extent of the trauma and the age of the patient. The clinical scenarios can rage from a minimal deciduous tooth structural loss on a young individual, to a facial bone fracture involving the esthetic area on an adult [14]. The treatment approaches would be completely different. The dental trauma on deciduous teeth could be seen as more favorable, first for the extent and, second, because primary teeth will be replaced by permanent ones. The loss of dentin and enamel could be adjusted with modern restorative procedures or with the use of temporary crowns [14]. The loss of an element could be replaced with a crown, adhered to neighboring teeth, such as Maryland bridges [14], or with the use of mini implants [57,58]. These options represent an increase on invasiveness, starting from a restorative solution to a surgical one. In this manuscript, the authors listed the surgical techniques that could be employed in medium to severe dental or facial injuries. If the trauma involves an adolescent, the two main options could be tooth transplantation and use of mini-implants. Both these solutions could be considered temporary or permanent. In case of auto-transplant, the tooth may still require root canal, final crown to match the esthetics and orthodontic treatment. Mini-implants can be retained for an extended period of time, and they would be considered final solutions. In the majority of cases, they are replaced by standard dental implants, upon adolescent end of growth [61]. In the most advanced cases, where teeth and alveolar bone had been lost during the sport accident, an extensive bone regeneration could be mandatory before dental implant therapy [61]. Besides the advantage of natural smile appearance, fixed type restoration and sparing of dental structure for prosthetic abutments, the standard implant therapy may not be well accepted because the length of treatment, the discomfort associated with surgery as well as the economic expense [61].

## 6. Conclusions

The high prevalence of TDI during sport activities can be a burden for the professional and amateur athletes. The likelihood of facial and dental injury poses the dental profession on the first line to treat damages that can alter and compromise patient’s function and esthetics. In particular, surgeons would be required in cases of avulsion, tooth, soft and hard tissue loss. In conclusion:

- The timing or tooth replantation after a traumas is crucial. All avulsed permanent and mature teeth eventually develop pulp necrosis. When the tooth is immature and presents an open apex, further root development can be achieved.
- The advances of dental CBCT and 3D printing allow the surgeon to successfully plan and execute tooth auto transplantations. The use of printed replica significantly decreased the surgical time and increased to more than 95% the survival rates.
- The soft and hard tissue deficiencies following trauma should be re-evaluated after initial healing and stabilization. The defects then can be treated as clinical scenario requiring standard soft and hard tissue regeneration.
- If the injured is an adult, tooth/teeth loss, tooth/teeth anxylosis and auto transplantation failure may be treated with dental implants to restore the missing tooth/teeth;
- The diffusion of injuries among adolescents calls for individualized treatment based on growth and time that separates the traumatic event to final restorations. Mini-implants could be used as an interim or final restoration to replace avulsed teeth.

## Figures and Tables

**Table 1:** List of traumatic dental injuries related to teeth and periodontal structures. Adapted from Levin L. et al. [15] and Bourguignon, C. et al. [16].

	TDI	Definition
Uncomplicated crown fractures	Enamel infraction	An incomplete fracture (crack or crazing) of the enamel, without loss of tooth structure
	Enamel fracture	A coronal fracture involving enamel only, with loss of tooth structure
	Enamel/dentin fracture	A fracture confined to enamel and dentin without pulp exposure
Complicated crown fractures	Enamel/dentin fracture with pulp exposure	A fracture confined to enamel and dentin with pulp exposure
Crown/root fracture		UNCOMPLICATED (WITHOUT PULP EXPOSURE) A fracture involving enamel, dentin, and cementum (note: crown-root fractures typically extend below the gingival margin) COMPLICATED (WITH PULP EXPOSURE) A fracture involving enamel, dentin, cementum, and the pulp (note: crown-root fractures typically extend below the gingival margin)
Root fractures		A fracture of the root involving dentin, pulp and cementum. The fracture may be horizontal, oblique or a combination of both
Alveolar fracture		The fracture involves the alveolar bone and may extend to adjacent bones
Concussion		An injury to the tooth-supporting structures without abnormal loosening or displacement of the tooth, but with marked reaction to percussion
Subluxation		An injury to the tooth-supporting structures with abnormal loosening, but without displacement of the tooth
Luxation	Extrusion	Displacement of the tooth out of its socket in an incisal/axial direction
	Lateral luxation	Displacement of the tooth in any lateral direction, usually associated with a fracture or compression of the alveolar socket wall or facial cortical bone
	Intrusion	Displacement of the tooth in an apical direction into the alveolar bone
Avulsion		Complete displacement of the tooth out of its socket

**Table 2:** Prevalence of traumatic dental injuries for permanent and primary teeth around the world. On the first column, sport and physical activity are reported as the causes of injuries. The last column indicates the number of studies used for this data. Adapted from “Textbook and Color Atlas of Traumatic Injuries” [14].

Cause	N Subjects	Prevalence	95% CI	N Studies
Primary and permanent teeth				
Sports	13,534	12.5%	8.2%–17.7%	21
Physical activity	10,481	19.45%	12.6%–27.3%	15
Permanent teeth				
Sports	4811	12.9%	8.3%–18.3%	14
Physical activity	2948	20.8%	14.0%–28.6%	8
Primary teeth				
Sports	1281	5.8%	3.2%–9.2%	6
Physical activity	1755	11.6%	2.8%–25.4%	9

**Table 3:** Treatment of tooth avulsion. Follow up regimens in weeks, months and years are listed, as well as the possible treatment options according to the different scenarios of tooth replanted at the site of injury, dry time of less or more than 60 min. S = SPLINT REMOVAL; R = RADIOGRAPH ADVISED EVEN IF NO CLINICAL SIGNS OR SYMPTOMS; RCT = root canal treatment; Adapted from Levin L et al. [15] and Bourguignon, C et al. [16].

	PERMANENT DENTITION												
	Follow-Up Regimens										Treatment		
Avulsion	**TDI**	1 W	2 W	4 W	6–8 W	3 M	4 M	6 M	1 Y	Yearly (at Least 5 y)	Tooth replanted at the site of injury or before the patient’s arrival at the dental clinic	Tooth kept in physiologic solution or non-physiologic conditions extra-oral dry time < 60 min.	Extra-oral dry time > 60 min
	Common treatment for mature and immature teeth										Clean injured area with water, saline, or chlorhexidine. Verify correct position of the replanted tooth clinically and radiographically. Leave the tooth/teeth in place (except if tooth is mal-positioned. Administer local anesthesia, if necessary, and preferably with no vasoconstrictor. If the teeth were replanted in the wrong socket or rotated, consider repositioning the tooth/teeth into the proper location up to 48 h after the traumatic incident. Stabilize tooth for 2 weeks using a passive flexible splint such as wire of a diameter up to 0.016” or 0.4 mm. Keep the composite and bonding agents away from the gingival tissues and proximal areas. Alternatively, nylon fishing line (0.13–0.25 mm) can be used to create a flexible splint. Nylon splints are not recommended for children when there are only a few permanent teeth for stabilization. in cases of associated alveolar fracture, a more rigid splint is indicated and should be left in place for about 4 weeks. Suture gingival lacerations, if present. Administer systemic antibiotics. Check tetanus status.	If visible contamination, rinse the root surface with saline or osmolality-balanced media to remove gross debris. Remove any debris by gently agitating it in the storage medium. Administer local anesthesia, preferably without a vasoconstrictor. Irrigate the socket with sterile saline. If there is a fracture of the socket wall, reposition the fractured fragment into its original position. Removal of the coagulum with a saline stream may allow better repositioning of the tooth. Replant the tooth slowly with slight digital pressure. Verify the correct position of the replanted tooth both clinically and radiographically. Stabilize tooth for 2 weeks using a passive flexible splint such as wire of a diameter up to 0.016” or 0.4 mm. Keep the composite and bonding agents away from the gingival tissues and proximal areas. Alternatively, nylon fishing line (0.13–0.25 mm) can be used to create a flexible splint. Nylon splints are not recommended for children when there are only a few permanent teeth for stabilization. in cases of associated alveolar fracture, a more rigid splint is indicated and should be left in place for about 4 weeks. Suture gingival lacerations, if present. Administer systemic antibiotics. Check tetanus status.	Remove loose debris and visible contamination by agitating the tooth in physiologic storage medium, or with gauze soaked in saline. Administer local anesthesia, preferably without vasoconstrictor. Irrigate the socket with sterile saline. If there is a fracture of the socket wall, reposition the fractured fragment. Replant the tooth slowly with slight digital pressure. The tooth should not be forced back to place. Verify the correct position of the replanted tooth both clinically and radiographically. Stabilize tooth for 2 weeks using a passive flexible splint such as wire of a diameter up to 0.016” or 0.4 mm. Keep the composite and bonding agents away from the gingival tissues and proximal areas. Alternatively, nylon fishing line (0.13–0.25 mm) can be used to create a flexible splint. Nylon splints are not recommended for children when there are only a few permanent teeth for stabilization. in cases of associated alveolar fracture, a more rigid splint is indicated and should be left in place for about 4 weeks. Suture gingival lacerations, if present. Administer systemic antibiotics. Check tetanus status.
	Avulsion (immature tooth)		S R	R	R	R		R	R	R	Initiate RCT within 2 weeks after replantation		
	Avulsion (mature tooth)		S R	R		R		R	R	R	Pulp revascularization, which can lead to further root development, is the goal when replanting immature teeth in children. The risk of external root resorption should be weighed against the chances of revascularization. If spontaneous revascularization does not occur, apexification, pulp revitalization/ revascularization, or root canal treatment should be initiated as soon as pulp necrosis and infection is identified		

## Data Availability

Data sharing not applicable. No new data were created or analyzed in this study. Data sharing is not applicable to this article.
